# The Synergies Between Understanding Belief Formation and Artificial Intelligence

**DOI:** 10.3389/fpsyg.2022.868903

**Published:** 2022-04-11

**Authors:** Sara Lumbreras

**Affiliations:** Institute for Research in Technology, Universidad Pontificia Comillas, Madrid, Spain

**Keywords:** artificial intelligence, belief, machine bias, complexity, reinforcement learning

## Abstract

Understanding artificial intelligence (AI) and belief formation have interesting bidirectional synergies. From explaining the logical derivation of beliefs and their internal consistency, to giving a quantitative account of mightiness, AI still has plenty of unexploited metaphors that can illuminate belief formation. In addition, acknowledging that AI should integrate itself with our belief processes (mainly, the capacity to reflect, rationalize, and communicate that is allowed by semantic coding) makes it possible to focus on more promising lines such as Interpretable Machine Learning.

## Introduction

The research program “Credition” has provided a solid framework to understand the phenomenon of believing and belief formation.

“*Credition, the processes of believing, are fundamental brain functions that enable a non-human animal or human being to trust his/her inner probabilistic representation(s). Credition is based on neural processes, including perception and valuation of objects and events in the physical and social environment in secular as well as religious contexts. By predictive coding, credition guides one’s actions and behaviors through reciprocating feedback involving learning* (Angel et al., 2017).”

Artificial Intelligence (AI) can be understood as a sequence of algorithms (that is, the mechanical application of some predefined steps) that is applied to a set of data and that generates a probabilistic representation of these data with the aim of making predictions, inferring a consequence or selecting the best possible option. AI can be understood as a machine that supports the formation and valuation of beliefs in the human and can be understood metaphorically as a belief-machine itself.

Artificial intelligence encompasses techniques such as clustering or pattern recognition. However, this paper focuses primarily on *Reinforcement Learning* (RL), where intelligent agents take actions in an environment to maximize a reward and punish mistakes. In RL, there are steps that are metaphorically described as perception (collecting new data), decision (selecting the optimal action), valuation (evaluating the outcome of a decision), or learning (the successive improvements in valuation obtained through repeated cycles of decision and valuation). For instance, a RL system could learn to play chess through cycles of selecting a move and valuing the possible positions.

For this reason, it is extremely interesting to examine AI from the lens of the credition process: understanding how AI works can give us insights to inform our hypothesis about the workings of credition. In parallel, acknowledging that AI should support belief formation helps to design it better and make this support as effective as possible.

## Synergies From Understanding Artificial Intelligence to Understanding Belief Formation

**FIGURE 1:**
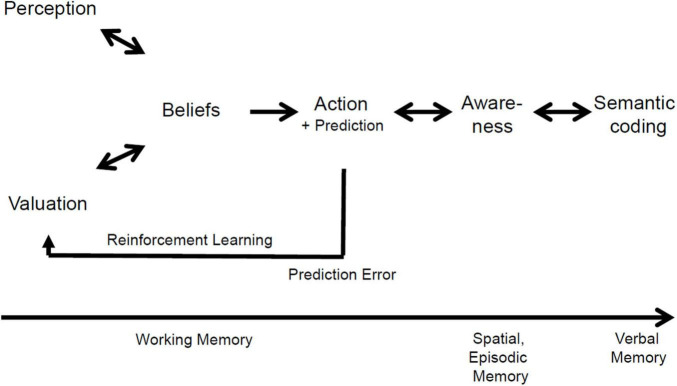
Schematic representation of a RL process in the context of credition. Source: Seitz et al. (in press), reproduced with permission.

Figure 1, taken from Seitz et al. (in press), presents a schematic representation of how a RL process can be understood in the context of credition, displaying the different levels of memory that are at play (Rolls, 2000). The inner probabilistic representation (which we refer to as “belief”) is built around the data received (perception) and is also used to value future actions (valuation). This representation is used to select the preferred course of action and predict its outcome. When new data are collected about the outcome (the *prediction error*), the probabilistic representation is updated through a new valuation in the process of RL. It is important to note that, according to this model, the credition process happens in a spontaneous and automatic manner, below the level of awareness.

For instance, one particularly interesting insight from the Creditions model and RL is the interpretation of the balance of *exploration* (which, in this context, we will understand the examination of new decisions or beliefs not tested before) vs. *exploitation* (the use of the existing belief system to make a decision in an efficient manner). This balance, which is key to the performance of RL algorithms, can be seen as an essential feature of belief formation and update that depends greatly on the psychological characteristics of each individual. In addition, In RL, each new data point is integrated into the probabilistic representation of the world. The specific manner in which this is done can be represented as an error minimization strategy. This model could be tested in experimental settings to improve our current understanding of how beliefs are formed and updated.

In addition, there also are interesting insights outside RL that can help us improve our understanding of the mechanisms of credition.

### Illuminating Metaphors From Artificial Intelligence Outside Reinforcement Learning

Reinforcement learning does not capture all the complexities of belief formation and update. Importantly, RL takes a blind approach to the inner probabilistic representation and does not necessarily impose any internal consistency to beliefs. However, we do know that new beliefs are more easily accepted when they are consistent with prior ones (Fryer et al., 2019) or that cognitive dissonance is an unpleasant experience (Cooper, 2007). We also know that some beliefs are derived logically from others. This makes it beneficial to resort also to some AI tools that have an emphasis in this logical consistency, or on the logical derivation of consequences. These are inference engines and Bayesian networks.

### Inference Engines and the Logical Derivation of Belief

Inference engines are tools that apply predefined logical rules to a knowledge database (also previously defined) to derive new facts from already known ones. We refer the reader to Colmerauer (1990) for a good introduction to Prolog, one of the first and most widely used inference engines which name is derived from the expression “Propositional Logic,” which is the basis of its first version (Prolog I), which was later upgraded to include first-order logic (quantification) or fuzzy logic (Zadeh, 1988) (which allows for intermediate states between true and false). Inference engines work in a similar manner to generic theorem provers such as the well-known *Isabelle* (Paulson, 1994).

Inference engines apply the logical rules first to the facts contained in the knowledge database to derive an initial set of consequences. This is performed by examining every potential pair for a possible conclusion. Then, both the initial facts and the newly obtained consequences are combined again to generate a second round of consequences. This process is repeated iteratively until no more new facts can be derived.

At some level, human beings also scan new knowledge for possible consequences using inference rules (Rodriguez et al., 2016). We could even understand that the more iterations of the process need to be performed in order to find a consequence, the more obscured it will be. It should be useful to incorporate a metaphor of the inference engine to the creditions model to understand how logical consequences are derived from new beliefs at the step of the inner probabilistic construction.

### Bayesian Networks and the Internal Consistency of Belief

A Bayesian Network is a probabilistic graphical model that represents a set of variables and their conditional dependencies *via* a directed acyclic graph. This means that variables are connected always with a direction (there is one cause and one consequence, without any possible circularity). Bayesian networks are a very interesting tool for understanding the contributing factor for an event. For example, it can represent the probabilistic relationships between diseases and their symptoms, so we could calculate the specific probabilities that an observed symptom is due to a given disease. We refer the reader to Chen and Pollino (2012) for a good tutorial on this topic. We know that beliefs are not held in isolation but could rather be understood as a network (Friedkin et al., 2016). For this reason, including the remarkable understanding of networks and their relationships that AI has created with Bayesian Networks can be extremely interesting.

### Complexity Theory to Understand the Global Properties of Belief Systems

A complex system is a system composed of many elements in interaction. We can find complex systems in contexts as different as the global climate, social organizations or the metabolism within a cell (Mitchell, 2009). Very importantly, the algorithms that support the developments of AI are also complex systems.

Complex systems have distinct properties which are shared by belief systems, which include being goal-oriented, open to receiving information from the exterior, spontaneous order (with hierarchies and context appearing), adaptation, being difficult to predict, experiencing non-linear phenomena (for instance, it is much more difficult to change a belief than to form it). I refer the reader to my paper (Lumbreras and Oviedo, 2020) for a more detailed account of these properties and their implications.

### Understanding Mightiness in a Quantitative Manner

In addition to its object, a belief can be characterized by its certainty, which can be very aptly represented by the certainty level in fuzzy logic that is embedded in Bayesian networks. Moreover, there is also a second qualifier: mightiness, which refers to the intensity that is attributed to this emotion. AI can also help us to understand this mightiness at a quantitative level. There is a property of the variables involved in a prediction model that is called *importance.* The importance of a variable is a measure of how much this variable affects the final decision. Arguably, we feel more intensely the beliefs that more profoundly affect our own identity and actions.

## Discussion: From Understanding Belief to Creating Better Artificial Intelligence

As explained in the section “Introduction,” the synergies between understanding AI and understanding belief are bidirectional, so there are positive outcomes that can be expected if we introduce what we know about belief formation into the way we design and use AI.

There are two main issues that plague the applications of AI: overfitting and machine bias. We need to remember that AI extracts patterns from the data that it receives for training, so it deeply depends on these data.

In overfitting, the data provided to the machine is not enough to be able to generalize. Much like a student that, instead of understanding, learns examples by heart, the algorithm fails when a new situation is considered.

In the same way, it is possible that the data we present to the algorithm does not represent reality fairly. For instance, it has been well documented that some algorithms disfavor African Americans, for instance when calculating their probability of recidivism in crime with the objective of deciding whether to grant them parole (Hajian et al., 2016). This was due to the database that was used for training containing a higher proportion of African American recidivists.

The problem with overfitting and machine bias is that they are not easy to detect. Most applications of AI are designed as *black boxes*, so we only have access to their specific predictions for every case but not to any reasoning behind them. Without detailed analyses, for instance, it is not possible to determine that the prediction is based on race, age, sex, or any other discriminatory variable. This means that when black-box AI is used in a high-stake decision (such as granting parole), this can have disastrous consequences.

As I have presented, black-box AI only shares predictions, and this can have disastrous consequences in high-stake problems. However, there is an emerging field within AI, *Interpretable ML*, which does allow for the understanding of models and their dynamics. This makes it possible avoid the problems derived from overfitting and machine bias (Molnar, 2020). The basic idea behind Interpretable ML is that, for many problems, it is possible to create AI that is so simple that can be expressed in rules understood by a human, and yet result in accurate predictions. These models would be the opposite to black boxes, they are transparent decision rules that can be understood and discussed.

For instance, Rudin (2019) developed an alternative to the parole algorithm that was based only on prior violent crimes and age. This shows that it is possible to create AI that better fits the way we form beliefs and is a more efficient support for our decision making.

## Conclusion

Understanding AI and understanding belief formation have interesting bidirectional synergies. From explaining the logical derivation of beliefs and their internal consistency, to giving a quantitative account of mightiness, AI still has plenty of metaphors to illuminate belief. Potentially, these can be used to simulate belief systems and arrive to testable predictions.

In addition, acknowledging what our belief processes have that AI lacks (mainly, the capacity to reflect, rationalize and communicate that is allowed by semantic coding) makes it possible for us to focus on creating AI that can better support decisions such as Interpretable ML.

## Data Availability Statement

The original contributions presented in the study are included in the article/supplementary material, further inquiries can be directed to the corresponding author.

## Author Contributions

The author confirms being the sole contributor of this work and has approved it for publication.

## Conflict of Interest

The author declares that the research was conducted in the absence of any commercial or financial relationships that could be construed as a potential conflict of interest.

## Publisher’s Note

All claims expressed in this article are solely those of the authors and do not necessarily represent those of their affiliated organizations, or those of the publisher, the editors and the reviewers. Any product that may be evaluated in this article, or claim that may be made by its manufacturer, is not guaranteed or endorsed by the publisher.
